# Assessment of the Cerebroplacental Ratio and Amniotic Fluid Index in Term Appropriate-for-Gestational-Age Pregnancies for Prediction of Adverse Perinatal Outcomes

**DOI:** 10.7759/cureus.100464

**Published:** 2025-12-31

**Authors:** Priyanka Verma, Devyani Misra, Vandana Gautam, Shailja Bhamri, Smriti Agrawal, Bhanupriya Singh

**Affiliations:** 1 Department of Obstetrics and Gynaecology, Dr. Ram Manohar Lohia Institute of Medical Sciences, Lucknow, IND; 2 Department of Obstetrics and Gynaecology, King George's Medical University, Lucknow, IND; 3 Department of Obstetrics and Gynaecology, Hind Institute of Medical Sciences, Barabanki, Barabanki, IND; 4 Department of Radiodiagnosis, Sanjay Gandhi Postgraduate Institute of Medical Sciences, Lucknow, IND

**Keywords:** amniotic fluid index, cerebroplacental ratio, doppler ultrasonography, perinatal risk assessment, term fetal monitoring

## Abstract

Background: Determining fetuses at risk of adverse perinatal outcomes at term remains a clinical challenge, particularly among pregnancies that appear low risk and appropriately grown for gestational age (AGA). While the cerebroplacental ratio (CPR) and amniotic fluid index (AFI) are established non-invasive indicators of fetal well-being, their combined predictive ability in low-risk AGA pregnancies is not well defined.

Objective: To evaluate and compare the predictive value of AFI alone and AFI combined with CPR in forecasting adverse perinatal outcomes among term AGA pregnancies.

Methods: This prospective observational study included 236 term AGA pregnancies, spanning 37 to 40 weeks of gestation. Participants were randomly assigned to two groups: Group 1 (AFI + CPR) and Group 2 (AFI only). Maternal characteristics, intrapartum findings, and neonatal outcomes were recorded. Statistical analyses, including receiver operating characteristic (ROC) curves, were used to assess the predictive performance of AFI and CPR for low Apgar (appearance, pulse, grimace, activity, respiration) scores, neonatal intensive care unit (NICU) admissions, and caesarean delivery.

Results: Baseline maternal and fetal characteristics were comparable between the groups. Normal vaginal delivery was the predominant mode of delivery, with no significant difference in neonatal morbidity or caesarean section rates between groups. ROC analysis demonstrated poor discriminatory ability of both AFI and CPR in predicting low Apgar scores, NICU admissions, or caesarean delivery. However, both parameters exhibited high negative predictive values, indicating utility in ruling out adverse outcomes. The addition of CPR did not significantly improve diagnostic accuracy compared to AFI alone.

Conclusion: In low-risk term AGA pregnancies, AFI remains a practical and widely used component of routine fetal surveillance, primarily reflecting its high negative predictive value rather than strong discriminatory capability for adverse outcomes. The inclusion of CPR does not provide a significant incremental benefit in predicting adverse perinatal outcomes, as both parameters demonstrate poor discriminatory performance. These findings support the role of AFI and CPR in excluding adverse outcomes rather than in predicting fetal compromise.

## Introduction

A major clinical challenge in obstetric practice is the identification of fetuses at risk of adverse perinatal outcomes at term, as the majority of perinatal morbidity and mortality occur in pregnancies traditionally classified as low risk [1]. Despite advances in antenatal surveillance, unanticipated fetal compromise continues to occur in women without identifiable risk factors, highlighting limitations in standard assessment tools. Reliance on fetal size alone is insufficient, as appropriately grown fetuses (AGA) may still exhibit subtle growth deceleration, impaired placental reserve, or reduced oxygen transfer [2]. Even in otherwise uncomplicated pregnancies, such subclinical alterations may contribute to intrapartum distress and adverse neonatal outcomes [3].

Conventional surveillance methods, including fetal biometry, cardiotocography (CTG), and assessment of amniotic fluid volume (AFV), provide useful information but often detect compromise late in its course due to limited specificity [4]. Doppler velocimetry has therefore become integral to modern antenatal monitoring, enabling dynamic evaluation of fetoplacental and cerebral circulation [5]. Importantly, Doppler abnormalities may be present even in fetuses with growth parameters within normal limits, reflecting early hemodynamic adaptation.

The cerebroplacental ratio (CPR), calculated as the ratio of the middle cerebral artery pulsatility index (MCA-PI) to the umbilical artery pulsatility index (UA-PI), is a key Doppler marker of fetal adaptation to hypoxia [6]. A reduced CPR reflects cerebral vasodilatation and increased placental resistance, consistent with a brain-sparing response. Low CPR values have been associated with adverse outcomes, including low Apgar (appearance, pulse, grimace, activity, respiration) scores, meconium-stained liquor (MSL), emergency caesarean delivery, neonatal intensive care unit (NICU) admission, abnormal fetal heart rate patterns, and perinatal mortality [7]. Notably, changes in CPR may precede other signs of fetal compromise, suggesting potential value as an early marker of intrapartum risk [8].

Amniotic fluid index (AFI) remains an essential component of prenatal assessment and is an indirect marker of chronic uteroplacental function and fetal renal perfusion [9]. Normal AFI ranges from 5 to 25 cm, with oligohydramnios (AFI <5 cm) reflecting placental insufficiency or chronic hypoxia, and polyhydramnios (AFI >25 cm) associated with fetal or maternal pathology [10]. Abnormal AFI values have been linked to increased risks of fetal distress, operative delivery, MSL, low Apgar scores, and perinatal mortality, making AFI a widely used and cost-effective surveillance tool in both low- and high-risk pregnancies [11].

Although both CPR and AFI have demonstrated independent associations with adverse perinatal outcomes, their combined predictive value in low-risk term AGA pregnancies remains uncertain. Most previous studies have focused on high-risk or growth-restricted populations, where Doppler abnormalities are more pronounced [12]. In contrast, evidence in AGA pregnancies is mixed, with some studies suggesting added prognostic value of CPR over AFI, while others report limited benefit [13]. These discrepancies may reflect differences in study design, timing of Doppler assessment, and applied cutoff thresholds.

Term gestation represents a critical transition period during which placental efficiency may decline, and even subtle impairments in placental function can increase susceptibility to intrapartum hypoxia and neonatal morbidity [14]. Evaluating both chronic placental reserve (AFI) and acute circulatory adaptation (CPR) may theoretically improve identification of fetuses at risk, although the clinical utility of such a combined approach in low-risk AGA pregnancies remains to be clearly established [15].

Objectives of the study

The objective of this study is to evaluate the predictive value of the AFI and CPR, individually and in combination, for adverse perinatal outcomes in term AGA fetuses. Specifically, the study aims to determine whether the addition of CPR to AFI improves the prediction of outcomes, including emergency caesarean section, low Apgar scores, NICU admission, and perinatal mortality, thereby enhancing antenatal surveillance in low-risk pregnancies.

## Materials and methods

Study design and setting

The study was a prospective observational research over an 18-month period between October 2022 and April 2024 in one of the Northern Indian tertiary care units. Ethical approval was obtained from the Institutional Ethics Committee prior to the study. Between 37 and 40 weeks of pregnancy, pregnant women were assessed on fetal growth, AFI, and CPR through ultrasound and Doppler. Participants were followed until delivery, and relevant maternal and neonatal outcomes were recorded for subsequent review.

Study participants

Inclusion Criteria

The target population of the study was pregnant women who visited the tertiary care hospital and met the eligibility criteria. Women who had singleton births between 37 and 40 gestation weeks, whose fetuses were well-grown relative to gestational age (AGA), and who gave informed consent were included. The participants had to be in the antenatal period before the labour set in.

Exclusion Criteria

Women with a gestational age of less than 37 weeks or 40 weeks or more, those with multiple gestations, or those requiring immediate delivery by caesarean section were excluded. Additional exclusion criteria were clinically determined fetal growth restriction (FGR), oligohydramnios or polyhydramnios, preterm membrane rupture, or any chronic maternal illnesses antagonistic to encompassing diabetes, chronic high blood pressure, or known kidney disease.

Sample size

The estimated sample size was 236. It is a sample of 236 patients who were randomly allocated to two groups through simple random sampling, where 118 participants were found in each group (Group A and Group B). The estimation of sample size was made due to the availability of a previous study that tested CPR and AFI at term and used them as reference parameters. The current study assumed the same effect on the computation.

The formula used for sample size estimation was:

$\text {Sample size}(\mathrm{n})=\frac{Z_{\alpha / 2}^2 \times(\text {S})\times(1-\text {S})}{(\text {D})^2}$

where Z = 1.96 at a 95% confidence interval, D = precision at 7%, and S = sensitivity.

$N=\frac{(1.96)^2\times(0.525)\times(1-0.525)}{(0.07)^2}=196$

After accounting for an anticipated 20% loss to follow-up, the final sample size was 236. Of these, 118 participants were assessed using both the AFI and CPR, while the remaining 118 were assessed using the AFI alone.

Data collection

The self-designed data collection tool was used to collect data upon the giving of written informed consent. The gestational age was determined according to the last menstrual period (LMP) and, where feasible, verified by the same through the crown-rump length using the latest ultrasound photograph.

Ultrasound and Fetal Biometry

The ultrasonography was used to measure fetal biometry. The estimated fetal weight (EFW) was determined using the Hadlock formula, which includes determining the femur's length, head circumference, abdomen circumference, and biparietal diameter. EFW values between the 10th and 90th percentile of gestational age (AGA) were deemed suitable, whereas those below the 10th percentile were classified as small in relation to gestational age (SGA).

Grouping of Participants

Participants were divided into two groups: Group 1: AFI and CPR both assessed; Group 2: Only AFI assessed. Allocation to the two groups was done using a simple allocation sequence for ultrasound assessment purposes and not as part of an interventional or randomized clinical trial design. The study was therefore conducted as a prospective observational study, with no protocol-mandated alteration in obstetric management based solely on CPR values.

Clinical decisions regarding induction of labour, mode of delivery, and intrapartum management were made by the attending obstetric team according to standard institutional protocols and routine clinical judgment, independent of study group assignment. The CPR measurements were recorded for observational and analytical purposes only and were not intended to guide or mandate clinical interventions, thereby minimizing potential performance bias related to Doppler findings.

Assessment of AFI

The AFI was measured using the Phelan method, which also measured the four imaginary quadrants that made up the uterus. Each quadrant's largest vertical amniotic fluid pockets were measured, and the results were added together to determine the AFI. The measurements were taken in a supine position of the patient. Only those women whose AFI ranged between 5 and 25 cm were considered in the study.

Assessment of CPR

Doppler ultrasonography was used to assess the CPR using a Mindray system (Mindray Bio-Medical Electronics Co., Shenzhen, China) equipped with a 2.5 MHz transabdominal curvilinear transducer. The middle cerebral artery (MCA) insonation was less than 30 degrees in this cross-sectional image of the embryonic head at the circle of Willis. The umbilical artery (UA) Doppler was measured in a free loop of the umbilical cord, and pulsatility indices (PIs) were obtained by automated tracings of at least three consecutive waveforms, which were recorded without fetal breathing motions and uterine contractions.

MCA PI/UA PI was used to estimate the CPR. Only cases with a CPR greater than one were included, which was used as a pragmatic fixed cut-off to define normal CPR in this study. Although gestational age-specific CPR percentiles are considered standard in the literature, a single numerical threshold was chosen to maintain uniformity across the narrow gestational age range studied (37-40 weeks) and to reflect real-world clinical practice in resource-limited settings. The participants were also monitored until delivery, and patient case records were used to get the information about intrapartum events and neonatal outcomes.

Outcome measures

The main finding of the research was to establish the diagnostic role of CPR and AFI in predicting the outcome of perinatal and the incidence of adverse outcomes in the presence of normal AFI. Lower Apgar scores of fewer than seven minutes, the need to perform newborn resuscitation, and the infant's admission to the NICU were the secondary outcomes. An emergency lower-segment caesarean section was performed due to fetal distress, and instrumental delivery was undertaken.

Data analysis

The information was entered into a spreadsheet in Microsoft Excel (Microsoft Corp., Redmond, WA, USA), where the confidentiality of all the study subjects was maintained. The statistical analysis was conducted with IBM SPSS Statistics for Windows, Version 24 (Released 2016; IBM Corp., Armonk, New York, United States). The data were summarized by using descriptive statistics, such as frequencies, percentages, means, medians (interquartile range), and standard deviations. The level of statistical significance was tested at 5% level (p < 0.05). The categorical variables in Groups 1 and 2 were compared using the Pearson chi-square test, which tested whether the observed and expected frequencies were different in contingency tables. Continuous variables in both groups were compared using an independent t-test, where the difference in group means was evaluated after assuming equal variance, and the t-test was determined through the pooled variance formula.

## Results

Baseline characteristics of study participants

There were similarities between the two groups' clinical and demographic characteristics. Maternal age, body mass index, socioeconomic status, education level, obstetric history, and the related co-morbidities, including hypothyroidism and gestational diabetes mellitus, did not have any statistically significant differences. This demonstrates how comparable the two groups were at baseline (Table 1).

**Table 1:** Demographic and clinical characteristics AFI: amniotic fluid index, CPR: cerebroplacental ratio, BMI: body mass index, SES: socioeconomic status, Primi: primigravida (woman pregnant for the first time), Multi: multigravida (woman who has been pregnant more than once), Nullipara: woman who has never given birth, Multipara: woman who has given birth two or more times, GDM: gestational diabetes mellitus, MNT: medical nutrition therapy

Variable		Group 1 (AFI + CPR)	Group 2 (Only AFI)	P-value
Age (years)	18-25	46 (39.0%)	38 (32.2%)	0.549
	26-30	67 (62.7%)	74 (62.7%)	
	>30	5 (4.2%)	6 (5.1%)	
BMI	18.5-22.9	65 (55.1%)	66 (55.9%)	0.593
	23-24.9	53 (44.9%)	51 (43.2%)	
	≥25	0 (0.0%)	1 (0.8%)	
Education	Illiterate	18 (15.2%)	17 (14.4%)	0.337
	Primary education	36 (30.5%)	12 (10.16%)	
	Secondary education	16 (13.5%)	7 (5.9%)	
	High school	26 (22.0%)	16 (13.5%)	
	Intermediate	18 (15.2%)	49 (41.5%)	
	Graduate and above	4 (3.3%)	17 (14.4%)	
SES	Upper SES	5 (4.23%)	7 (5.9%)	0.574
	Upper middle SES	7 (5.9%)	4 (3.38%)	
	Lower middle SES	39 (33.0%)	24 (20.3%)	
	Upper lower SES	26 (22.0%)	45 (38.1%)	
	Lower SES	41 (34.7%)	38 (32.20%)	
Gravida	Primi	62 (52.5%)	58 (49.1%)	0.883
	Multi	56 (47.4%)	60 (50.8%)	
Parity	Nullipara	78 (66.1%)	70 (59.3%)	0.582
	Multipara	40 (33.8%)	48 (40.6%)	
History of surgery	Gynaecologic surgery	2 (ovarian cystectomy)	2 (ovarian cystectomy)	0.221
	Non-gynaecologic surgery	3 (1 appendectomy, 1 laparoscopy, 1 cholecystectomy)	3 (laparotomy)	
	No surgical history	113	113	
Co-morbidity	Hypothyroidism	19 (16.1%)	12 (10.2%)	0.095
	Cholestasis	3 (2.5%)	11 (9.3%)	
	GDM on MNT	11 (9.3%)	9 (7.6%)	
	No medical history	85	86	

Mode of delivery and labour findings

Normal vaginal delivery was the most common mode of delivery in both groups, with comparable rates of caesarean section. Labour onset characteristics differed between the groups, with spontaneous labour occurring more frequently in the AFI-only group, while a higher proportion of women in the AFI + CPR group underwent induction of labour (32.2% vs. 14.4%). In terms of intrapartum findings, the AFI-only group demonstrated a higher proportion of thin MSL, whereas thick MSL was more frequently observed in the AFI + CPR group. The distribution of delivery-related outcomes across the two groups is shown in Table 2.

**Table 2:** Mode of delivery and intrapartum findings among study participants AFI: amniotic fluid index, CPR: cerebroplacental ratio, CTG: cardiotocography, NA: not applicable

Variable	Group 1 (AFI + CPR)	Group 2 (AFI only)	P-value
Mode of delivery			
Normal vaginal delivery (NVD)	90 (76.2%)	89 (75.4%)	0.358
Lower segment caesarean section (LSCS)	26 (22.0%)	24 (20.3%)	
Instrumental delivery	2 (1.7%)	5 (4.2%)	
Indications for LSCS			
Meconium-stained liquor with fetal distress	7 (32.1%)	6 (33.3%)	0.363
Persistent category II CTG	4 (14.3%)	9 (33.3%)	
Non-progress of labour	6 (21.4%)	5 (18.5%)	
Meconium-stained liquor	8 (28.6%)	4 (14.8%)	
Second stage arrest	1 (3.6%)	0 (0.0%)	
Indication for instrumental delivery			
Meconium-stained liquor with fetal distress	2 (28.6%)	5 (71.4%)	NA
Intrapartum findings			
Adequate/clear liquor	2 (7.4%)	0 (0.0%)	0.019
Absent liquor	2 (7.4%)	1 (4.5%)	
Thin meconium-stained liquor	14 (51.9%)	18 (81.8%)	
Thick meconium-stained liquor	7 (25.9%)	2 (9.1%)	
Placental abruption	0 (0.0%)	2 (9.1%)	
Cord around the neck	1 (3.7%)	1 (4.5%)	

Neonatal outcomes

The two study groups shared nearly similar neonatal outcomes. No meaningful difference was observed in the neonatal unit (NNU) admissions, Apgar scores, and the necessity of resuscitation. In the AFI +CPR group, meconium aspiration was the most common reason to be admitted to the NNU, whereas respiratory distress syndrome (RDS) was found more in the AFI only group. Neonatal mortality differed slightly more in the AFI-only group, although it was not statistically significant (Table 3).

**Table 3:** Neonatal outcomes among study participants across both groups AFI: amniotic fluid index, CPR: cerebroplacental ratio, NNU: neonatal nursery unit, MSL: meconium-stained liquor, RDS: respiratory distress syndrome, Apgar: appearance, pulse, grimace, activity, respiration

Variable		Group 1 (AFI + CPR)	Group 2 (Only AFI)	P-value
NNU admission	Yes	8 (6.8%)	11 (9.3%)	0.526
	No	110 (93.2%)	107 (90.7%)	
Indication for NNU admission	MSL aspiration	6 (75.0%)	5 (45.5%)	0.198
	RDS	2 (25.0%)	6 (54.5%)	
Apgar score @ 1 min	<7	29 (24.6%)	27 (22.9%)	0.760
	≥7	89 (75.4%)	91 (77.1%)	
Need for resuscitation	Yes	12 (10.2%)	16 (13.6%)	0.421
	No	106 (89.8%)	102 (86.4%)	
Resuscitation outcome	Discharged	10	13	0.065
	Mortality	2 (Expired: Day 6 - RDS, Day 7 - MSL)	3 (Expired: Day 4 and 5 - MSL, Day 2 - RDS)	

Maternal outcomes

Both groups had similar and favourable maternal outcomes. Most of the women in both groups were sent home in good health, and the maternal deaths were minimal. The effect size of the difference in maternal outcome was not significant (Table 4).

**Table 4:** Comparison of maternal outcome in groups AFI: amniotic fluid index, CPR: cerebroplacental ratio, N: number

Outcome	Group 1 (AFI + CPR), N = 118	Group 2 (only AFI), N = 118	P-value
Discharged in satisfactory condition	116 (98.3%)	115 (97.5%)	0.651
Mortality	2 (1.7%)	3 (2.5%)	

Receiver Operating Characteristic (ROC) Analyses

**Figure 1:**
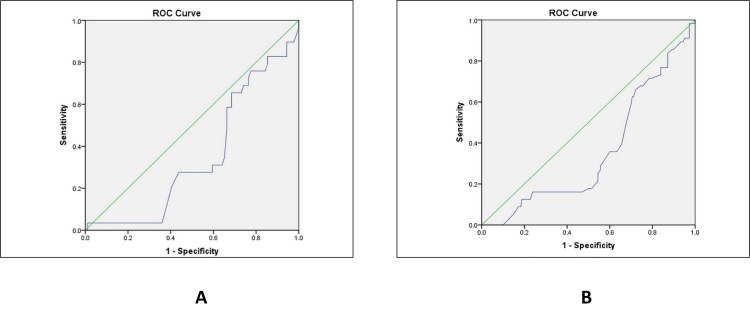
ROC analysis for predicting Apgar score ≤7 in (A) AFI + CPR and (B) AFI only ROC: receiver operating characteristic; AFI: amniotic fluid index; CPR: cerebroplacental ratio; Apgar: appearance, pulse, grimace, activity, respiration

ROC analysis demonstrated poor discriminatory ability of both AFI alone and AFI combined with CPR for predicting a low Apgar score (≤7). The area under the curve (AUC) for AFI combined with CPR was 0.351 (95% CI: 0.27-0.43), while the AUC for AFI alone was 0.358 (95% CI: 0.28-0.44). The CIs for both parameters were wide and approached values close to 0.5, indicating limited discriminatory performance. Although sensitivity and negative predictive values were numerically moderate, specificity was low, resulting in poor overall diagnostic accuracy. Consequently, neither parameter demonstrated clinically meaningful predictive ability for low Apgar scores (Figure 1).

Prediction of NNU admission

**Figure 2:**
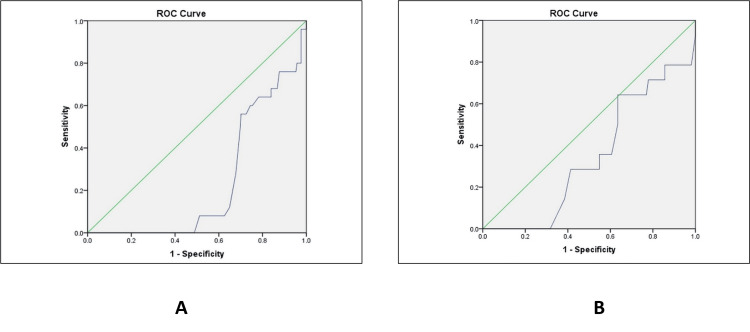
ROC analysis for predicting NNU admission in (A) AFI + CPR and (B) AFI only ROC: receiver operating characteristic; AFI: amniotic fluid index; CPR: cerebroplacental ratio; NNU: neonatal unit

For the prediction of NNU admission, both AFI alone and AFI combined with CPR similarly demonstrated poor discriminatory performance. Despite relatively high negative predictive values, the AUC remained low for both AFI (AUC = 0.237, 95% CI: 0.17-0.30) and AFI combined with CPR (AUC = 0.345, 95% CI: 0.26-0.43), reflecting limited overall discrimination. These findings suggest that while both parameters may be helpful in excluding adverse outcomes, they do not provide reliable predictive capability for identifying neonates requiring NNU admission (Figure 2).

Prediction of caesarean delivery (lower segment caesarean section (LSCS))

**Figure 3:**
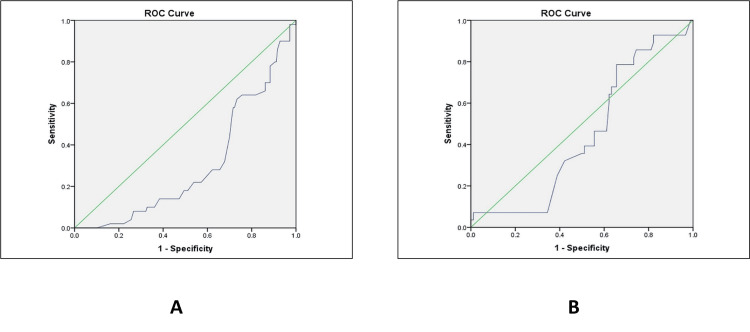
ROC analysis for predicting LSCS delivery in (A) AFI + CPR and (B) AFI only ROC: receiver operating characteristic; AFI: amniotic fluid index; CPR: cerebroplacental ratio; LSCS: lower segment caesarean section

In the case of caesarean delivery prediction, AFI showed a slightly higher AUC than AFI with CPR, but neither of them was statistically significant. There were relatively high sensitivity and low specificity in both groups, indicating low diagnostic accuracy in identifying women who would need LSCS (Figure 3).

## Discussion

AFV and CPR are established non-invasive antepartum parameters used to assess fetal well-being. While the AFI is a widely utilized indicator of chronic intrauterine stress, CPR has gained attention as a marker of adverse perinatal outcomes, particularly in pregnancies complicated by FGR. However, its role in AGA remains less clearly defined. The present study evaluated and compared perinatal outcomes in term AGA pregnancies assessed using AFI alone versus AFI combined with CPR. The study included 236 pregnant women with AGA fetuses, equally distributed between the two groups. The majority of participants were between 26 and 30 years of age, consistent with demographic patterns reported in similar populations [16].

No significant differences were observed between the two groups with respect to overall delivery outcomes. Normal vaginal delivery was the most common mode of delivery, and caesarean section rates were comparable between groups. However, induction of labour occurred more frequently in the AFI + CPR group (32.2%) compared with the AFI-only group (14.4%), reflecting differences in labour onset characteristics between the two groups. These findings are consistent with previous reports describing higher rates of operative delivery in pregnancies with abnormal AFI attributable to fetal distress [17], as well as studies reporting increased intervention in cases with abnormal CPR and non-reassuring fetal heart rate patterns [18].

MSL was commonly observed in both groups. Thin MSL was more prevalent in the AFI-only group, whereas thick MSL was more frequent in the AFI + CPR group. Occasional findings included a cord around the neck and placental abruption. The most frequent indication for caesarean section was MSL with fetal distress. These observations are in agreement with earlier studies demonstrating an association between low AFI and increased rates of MSL and fetal distress [19], although some studies have reported inconsistent associations [20]. Meta-analytic data have nonetheless confirmed a significant correlation between AFI ≤5 cm and increased rates of caesarean section for fetal distress [21].

ROC analysis demonstrated poor predictive performance of both AFI and CPR for caesarean section. AFI showed a low AUC (0.303, p = 0.001), characterized by high sensitivity but poor specificity. The addition of CPR did not significantly improve predictive performance (AUC = 0.447, p = 0.395), and overall diagnostic accuracy remained limited in both groups. These findings align with previous studies reporting weak predictive validity of AFI and CPR for operative delivery outcomes [22].

Neonatal outcomes, including NNU admission, Apgar scores, and resuscitation requirements, did not differ significantly between groups. Although NNU admissions were slightly more frequent in the AFI-only group, meconium aspiration and RDS were the most common indications. Low Apgar scores (≤7 at five minutes) and need for resuscitation were comparable across groups. These findings are consistent with earlier reports suggesting that while low AFI may be associated with increased neonatal morbidity, the addition of CPR provides limited incremental benefit in uncomplicated pregnancies [23].

The limited predictive ability of both parameters was further confirmed by ROC analyses. For prediction of low Apgar scores (≤7), AFI demonstrated an AUC of 0.358 (p = 0.001), while AFI combined with CPR yielded an AUC of 0.351 (p = 0.001). For NNU admission, the AUCs were 0.237 (p = 0.001) for AFI and 0.345 (p = 0.061) for AFI + CPR. Despite isolated sensitivity values appearing numerically moderate, the extremely low AUC values indicate poor discriminatory performance, in some instances approaching or falling below chance levels. Poor specificity further limited overall diagnostic accuracy. Consequently, the addition of CPR did not enhance the predictive performance of AFI for adverse perinatal outcomes in this cohort. These findings underscore that sensitivity alone, in the absence of adequate discriminatory ability as demonstrated by ROC analysis, should not be interpreted as evidence of clinically meaningful predictive utility.

Previous literature has reported conflicting results regarding the role of CPR in AGA pregnancies. Some studies have demonstrated associations between low CPR and increased rates of NICU admission, operative delivery, and adverse perinatal outcomes [24], with meta-analyses supporting relationships with low Apgar scores, MSL, and abnormal fetal heart rate patterns [25]. Conversely, other studies have reported limited benefit of combining CPR with AFI in predicting adverse outcomes [26,27]. The heterogeneity in findings may reflect differences in study populations, Doppler timing, and applied thresholds.

Although CPR theoretically reflects placental resistance and fetal circulatory adaptation, the present study demonstrates that its combined use with AFI in low-risk AGA pregnancies does not provide clinically meaningful improvement over AFI alone. The similarly limited diagnostic performance of both markers highlights the difficulty of detecting subtle placental compromise in uncomplicated pregnancies using current surveillance tools. The higher rate of labour induction observed in the AFI + CPR group represents a difference in obstetric management patterns rather than a protocol-driven intervention, as clinical decisions were made according to standard institutional practice.

Strengths and limitations

The strengths of this study include its prospective observational design, focus on a clinically relevant low-risk AGA population, and the use of simple, widely available, and reproducible ultrasound and Doppler techniques applicable to routine obstetric practice. The study addresses an important gap in the literature by evaluating both the individual and combined predictive performance of AFI and CPR in uncomplicated term pregnancies, which represent the majority of obstetric cases.

A limitation of the study is the assessment of CPR as a single Doppler parameter without the inclusion of additional indices, such as the UA-PI, which may further refine risk stratification. Nevertheless, this focused approach enabled evaluation of the incremental value of CPR over AFI within a pragmatic clinical framework.

## Conclusions

This prospective observational study demonstrates that, in low-risk appropriately grown term pregnancies, the addition of CPR assessment to AFI evaluation does not provide meaningful improvement in the prediction of adverse perinatal outcomes. Both parameters showed limited discriminatory ability for outcomes such as emergency caesarean section, NICU admission, and low Apgar scores, despite comparable diagnostic performance. While high negative predictive values indicate a role in excluding adverse outcomes rather than predicting compromise, CPR did not confer substantial incremental value over AFI alone. These findings add to the current evidence on intrapartum risk assessment at term and highlight the need for further prospective studies to better define the clinical contexts in which Doppler-based parameters may be informative.
